# Trophoblast inclusions and adverse birth outcomes

**DOI:** 10.1371/journal.pone.0264733

**Published:** 2022-03-01

**Authors:** Morgan R. Firestein, Harvey J. Kliman, Ayesha Sania, Lucy T. Brink, Parker H. Holzer, Katherine M. Hofmann, Kristin M. Milano, Nicolò Pini, Lauren C. Shuffrey, Hein J. Odendaal, William P. Fifer

**Affiliations:** 1 Department of Psychiatry, Columbia University Irving Medical Center, New York, New York, United States of America; 2 Department of Obstetrics, Gynecology and Reproductive Sciences, Yale University School of Medicine, New Haven, Connecticut, United States of America; 3 Faculty of Medicine and Health Science, Department of Obstetrics and Gynaecology, Stellenbosch University, Cape Town, Western Cape, South Africa; 4 Department of Statistics & Data Science, Yale University, New Haven, Connecticut, United States of America; 5 Department of Pediatrics, Columbia University Irving Medical Center, New York, New York, United States of America; 6 Division of Developmental Neuroscience, New York State Psychiatric Institute, New York, New York, United States of America; University of Wisconsin - Madison, School of Veterinary Medicine, UNITED STATES

## Abstract

**Objective:**

Trophoblast inclusions—cross sections of abnormal trophoblast bilayer infoldings—have previously been associated with aneuploidy, placenta accreta, and prematurity. This study was conducted to establish the relationship between trophoblast inclusions and a range of placental, pregnancy, and birth outcomes in a patient population with high smoking and alcohol exposure. Specifically, we sought to evaluate the association between the presence of trophoblast inclusions and 1) three primary birth outcomes: full-term birth, preterm birth, and stillbirth; 2) gestational age at delivery; and 3) specific placental pathologies.

**Methods:**

Two slides containing chorionic villi were evaluated from 589 placentas that were collected from Stellenbosch University in Cape Town, South Africa as part of the prospective, multicenter cohort Safe Passage Study of the Prenatal Alcohol and SIDS and Stillbirth Network. The subsample included 307 full-term live births, 212 preterm live births, and 70 stillbirths.

**Results:**

We found that the odds of identifying at least one trophoblast inclusion across two slides of chorionic villi was significantly higher for placentas from preterm compared to term liveborn deliveries (OR = 1.74; 95% CI: 1.22, 2.49, *p* = 0.002), with an even greater odds ratio for placentas from stillborn compared to term liveborn deliveries (OR = 4.95; 95% CI: 2.78, 8.80, *p <* 0.001). Gestational age at delivery was inversely associated with trophoblast inclusion frequency. Trophoblast inclusions were significantly associated with small for gestational age birthweight, induction of labor, villous edema, placental infarction, and inflammation of the chorionic plate.

**Conclusions:**

The novel associations that we report warrant further investigation in order to understand the complex network of biological mechanisms through which the factors that lead to trophoblast inclusions may influence or reflect the trajectory and health of a pregnancy. Ultimately, this line of research may provide critical insights that could inform both clinical and research applications.

## Introduction

Throughout pregnancy, the placenta serves as the critical interface between mother and fetus. Instances of abnormal development and functioning of the placenta have been implicated in maternal and fetal mortality and numerous morbidities including preeclampsia, maternal hemorrhage, preterm birth, and intrauterine growth restriction [1,2]. Therefore, careful examination of the human placenta is critical in understanding the etiology of many adverse pregnancy outcomes.

Typical placental development involves the fusion of mononuclear cytotrophoblast cells into the multinucleated syncytiotrophoblast layer [3]. When either the proliferation rate of the cytotrophoblast becomes excessive, or the fusion rate to form the syncytium decreases, the trophoblasts bilayer bends inwards forming trophoblast invaginations within the chorionic villi [4,5]. When cut in cross section, these invaginations appear as trophoblast inclusions (TIs), which have been identified as important indicators of abnormal placental development [6]. Previous studies have reported TIs in 93% of cases of aneuploidy [5] and in 47% of cases of placenta increta and percreta [7]. In addition to the substantial evidence suggesting that TIs may either be the result or an indicator of an abnormal karyotype, these abnormalities continue to be reported in placentas from karyotypically normal pregnancies. Importantly, the presence of TIs in karyotypically normal pregnancies has been associated with adverse birth and developmental outcomes. Specifically, TIs are observed in a greater proportion of placentas from preterm births [8] and at a higher frequency in placentas of children with autism and their siblings [9,10].

To gain deeper insight into the relationship between TIs and pregnancy, birth and placental pathologies, we performed a rigorous histologic examination on a subset of n = 589 placentas from the Safe Passage Study (SPS) of the Prenatal Alcohol and SIDS and Stillbirth (PASS) Network [11,12] to assess the association between the presence of TIs and three primary birth outcomes: full-term birth, preterm birth, and stillbirth—and the relationship between trophoblast inclusions and gestational age at delivery. Additionally, we aimed to determine whether trophoblast inclusions were associated with specific placental pathologies.

## Materials and methods

### Study participants

The SPS, a prospective, multicenter cohort study, enrolled n = 10,088 women (12,029 fetuses) during pregnancy between August 2007-October 2016 across multiple study sites in North Dakota and South Dakota in the United States, and in Cape Town, South Africa [11,12]. The data included in this analysis originated solely from Stellenbosch University in Cape Town, South Africa. All study procedures and analyses included here were reviewed and approved by the Stellenbosch University Health Research Ethics Committee (project #9317) and the New York State Psychiatric Institute Institutional Review Board (protocol #5338). Microscopic examination of the deidentified slides sent for evaluation to the Yale Reproductive and Placental Research Unit was approved by the Stellenbosch University Ethics Board and the Yale University Human Investigation Committee (protocol #1003006495). Informed written consent was obtained from all study participants.

Pregnant women were eligible to participate if they 1) were able to provide informed written consent; 2) were pregnant with one or two fetuses; 3) were ≥16 years of age; 4) had a gestational age (GA) of at least 6 weeks + 0 days and were not at the delivery admission unit; and 5) were fluent in English or Afrikaans.

To accommodate simultaneous sub-analyses of the data, slides were requested and retrieved for n = 594 placentas based on one or both of the following criteria: 1) the pregnancy resulted in a preterm live birth or a stillbirth between 20 weeks + 0 days and 43 weeks GA (n = 282) or 2) the pregnancy resulted in a full-term live birth and the infant subsequently participated in at least one follow-up assessment between birth and 4 years of age (n = 307). Of the 594 births included in this sub-analysis, there were 576 singleton pregnancies and 9 sets of twins.

### Tissue collection, preparation, and examination

Tissue was collected from 28% of the participants as part of an embedded study [11]. Whole placentas were collected at the time of delivery and refrigerated at 4°C until processing, usually the next working day. After trimming the membranes and umbilical cord, 1–2 cm transverse cuts were made through the maternal surface. Two blocks of tissue were obtained from cross-sections of normal parenchyma and placed into a cassette. Placental blocks were fixed in 10% neutral buffered formalin, stored at room temperature, and paraffin wax blocks were prepared. Two hematoxylin and eosin stained slides were produced for histological examination of each placenta.

**Fig 1:**
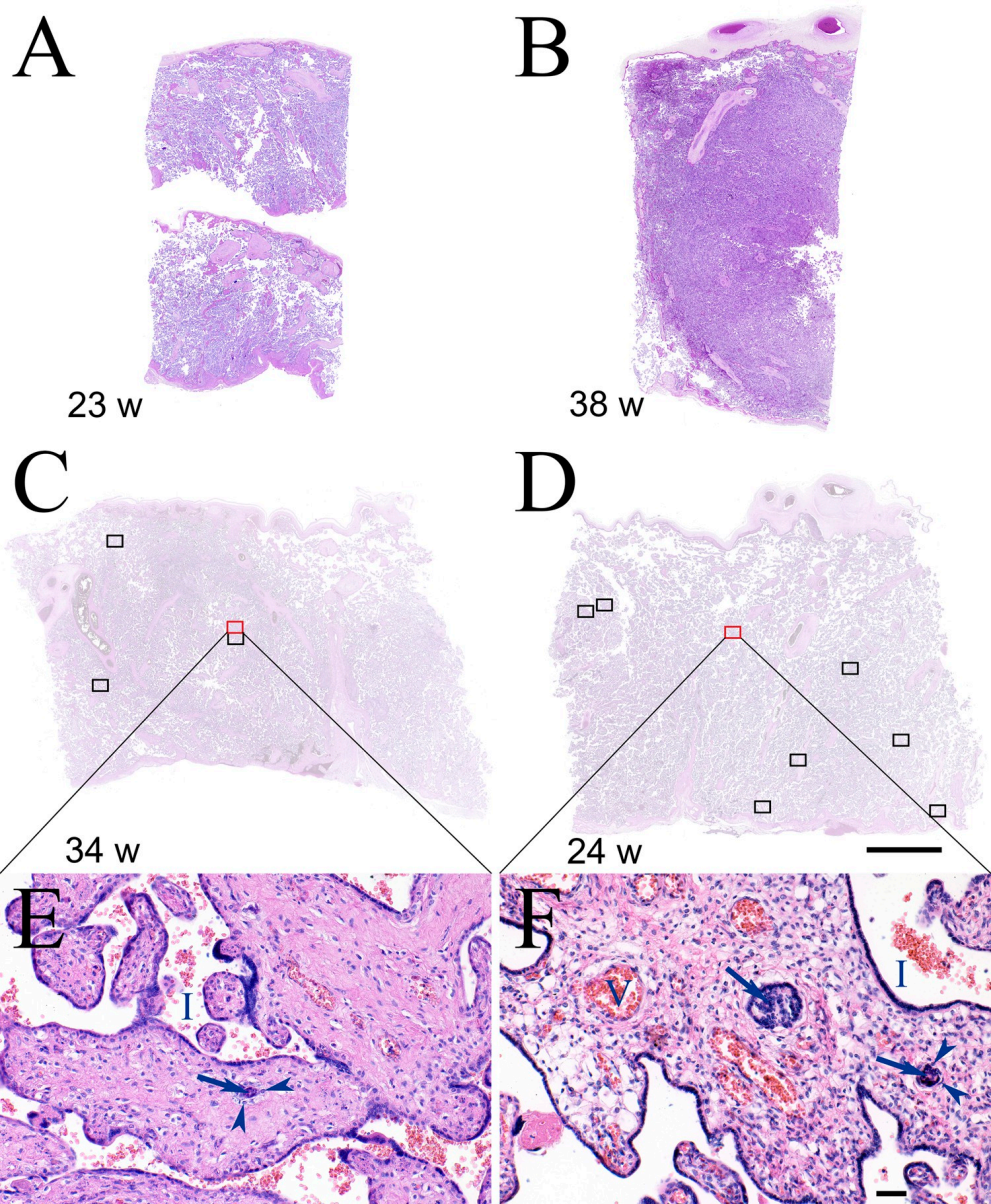
Representative villi from none, mild and marked TI categories. A) A slide from a representative case at 23 weeks for which no TIs were identified. B) A slide from representative case at 38 weeks for which no TIs were identified. C) A slide from a representative case at 34 weeks that was in the ‘mild’ TI category. Four TIs were identified in this slide (indicated by rectangles) and an additional 3 TIs were identified in the second slide (not shown) from this case (average 3.5/slide = ‘mild’ category). The villi within the red rectangle are enlarged in panel E which demonstrates a single TI with central syncytiotrophoblast nuclei (arrow) surrounded by cytotrophoblasts (arrow heads). Intervillous space (I). D) A slide from a representative case at 24 weeks that was in the ‘marked’ TI category. Nine TIs were identified in this slide (indicated by rectangles) and an additional 10 TIs were identified in the second slide (not shown) from this case (average 9.5/slide = ‘marked’ category). The villi within the red rectangle are enlarged in panel F which demonstrates two TIs in one villus with central syncytiotrophoblast nuclei (arrows) surrounded by cytotrophoblasts (arrow heads). Intervillous space (I). Panels A-D are all at the same magnification, scale bar = 5 mm. Panels E and F are at the same magnification, scale bar = 50 μM.

The two de-identified slides from each placenta were evaluated at the Yale Reproductive and Placental Research Unit. The 1,188 slides were randomized and re-labeled in numeric order and were blindly read by a placental pathologist (HJK). The pathologist was blind to all demographic, clinical, prenatal, birth outcome, and developmental outcome data. All slides were examined to determine the frequency of TIs. Based on previous methods, we determined the total number of inclusions and calcified inclusions [6,8,10]. Using this same dataset, we previously identified four TI subtypes and determined that they were highly inter-related [6]. In the present analysis, we separately quantitated the number of each subtype and collapsed across the four subtypes to calculate the total number of TIs per slide. The average number of TIs per slide for each placenta was calculated by adding the total number of TIs per pair of slides and dividing by 2 (the number of slides from each placenta). The average number of TIs per slide was then assigned into one of three categories; ‘none’ (average of 0 TIs), ‘mild’ (average of >0–5 TIs), or ‘marked’ (average of >5 TIs) (Fig 1). In addition, each slide was assessed for a broad range of placental pathologies. Intra-rater test-retest reliability of 81.5%-94.1% and an adjusted percent agreement of 99.2%-100% was established [6]. Slides from five placentas were excluded due to poor fixation, resulting in a final sample of n = 589 placentas (9 twin pregnancies).

### Classification of primary birth outcomes

Pregnancy and birth outcomes were characterized in detail as part of the SPS study [11,12]. Medical records were abstracted and births was categorized into one of three groups: i) full-term livebirth (FTLB), defined as a live birth at or after 37 weeks GA; ii) preterm livebirth (PTLB), defined as a live birth before 37 weeks GA; and iii) stillbirth (SB), defined as fetal death at or after 20 weeks GA.

### Classification of birth characteristics and placental pathology

Birth characteristics and measures of placental pathology were determined using data obtained as part of the original SPS study and through histological examination of the slides. From the original study records, we obtained the mode of delivery (vaginal versus cesarean delivery), labor onset (spontaneous versus induced), GA at delivery (determined by prenatal ultrasound), and birth weight relative to GA. Using the trimmed weights for all placentas collected through the SPS study (n = 1556), we generated growth curves of placental weight for GA at delivery separately for males and females [13]. Placentas included in the present analysis were then categorized as being small for GA (<10^th^ percentile), appropriate for GA (10^th^-90^th^ percentile), or large for GA (>90^th^ percentile). As part of our pathologic examination, we recorded the absence or presence of villous edema (the swelling of chorionic villi with interstitial fluid in the villus core) [14], the absence or presence of any stage of maternal inflammation (neutrophils) in the chorionic plate (a marker of the maternal inflammatory response following an ascending infection) [15], the percentage of infarction (the death of tissue) [16], the absence or presence of a fetal inflammatory response (neutrophilic migration through the fetal chorionic plate vessels) [15], increased syncytial knots (evidence of premature maturation of the placenta), chronic villitis (evidence of a maternal immunologic reaction against the placenta) [17], increased intervillous fibrin, subchorionic plate hematoma, or increased subchorionic plate fibrin (all showing evidence of decreased maternal perfusion of the placenta) [18], or the absence or presence of meconium laden macrophages in the chorionic plate (evidence of intrauterine defecation) [19].

### Statistical analysis

We used descriptive statistics, mean and standard deviation for continuous variables, count and percentages for categorical variables to describe the characteristics of the study population by birth outcome groups. We used logistic regression models to estimate the odds ratio and 95% confidence intervals of presence of TI associated with birth outcomes. Separate models were fit for each birth outcome. Models were adjusted for maternal age at delivery, education status, marital status, parity, maternal height, and prenatal depression as potential confounders. A missing indicator method was used in multivariate models when a covariate was missing. To determine whether the association between TIs and birth outcome was dependent on GA at delivery, we compared placentas from livebirths to those from stillbirths and performed a linear regression model that included GA at delivery as a covariate. We used separate Univariate logistic regression models to examine the association of TIs with birth characteristics and placental pathologies. All analyses were performed in SAS software version 9.4 (SAS Institute, Cary NC) and Python packages of statsmodels and Matplotlib.

## Results

### Study sample characteristics

The study sample included n = 307 FTLBs, n = 212 PTLBs, and n = 70 SBs (all of which were delivered <37 weeks GA). Demographic and clinical characteristics for the overall sample and each birth outcome group are presented in Table 1. Across the entire sample, the majority of the pregnancies were multiparous (64.7%) and resulted in a vaginal delivery (86.4%). GA at delivery ranged from 20.9 to 43.0 weeks with a mean of 36.0 weeks (SD = 4.82). Among pregnancies for which sex could be determined, 51.5% were male and 48.5% female. The majority of women were between 20–34 years of age at the time of delivery (76.2%), self-identified as “other or mixed race” (99.7%) (not shown), and had completed some high school (69.1%). About half the sample was married/living with a partner (48.2%). Maternal height was considered as it is a marker of long-term nutrition [20], and the majority of the women were taller than 155 cm (63.8%). In the overall sample, 48.0% of women scored in the ‘low risk’ range of the Edinburg Postpartum Depression Scale.

**Table 1:** Demographic and clinical characteristics.

Variable No. (%) or Mean (SD)	Overall (N = 589)	Full-Term (N = 307)	Preterm (N = 212)	Stillbirth (N = 70)
**Maternal Education Attainment**				
Any Primary School	37 (6.3%)	15 (4.9%)	15 (7.1%)	7 (10.0%)
Some High School	407 (69.1%)	207 (67.4%)	151 (71.2%)	49 (70.0%)
Completed High School	113 (19.2%)	67 (21.8%)	34 (16.0%)	12 (17.1%)
Beyond High School	30 (5.1%)	18 (5.9%)	10 (4.7%)	2 (2.9%)
Declined or Unknown	2 (0.3%)	0 (0%)	2 (0.9%)	0 (0%)
**Maternal Employment Status**				
Employed	418 (71.0%)	98 (31.9%)	57 (26.9%)	16 (22.9%)
Unemployed	171 (29.0%)	209 (68.1%)	155 (73.1%)	54 (77.1%)
**Marital Status**				
Not Married	305 (51.8%)	153 (49.8%)	114 (53.8%)	38 (54.3%)
Married	284 (48.2%)	154 (50.2%)	98 (46.2%)	32 (45.7%)
**Maternal Height (cm)**				
< 145 cm	15 (2.5%)	10 (3.3%)	3 (1.4%)	2 (2.9%)
145–149 cm	45 (7.6%)	26 (8.5%)	13 (6.1%)	6 (8.6%)
150–155 cm	153 (26.0%)	68 (22.1%)	64 (30.2%)	21 (30.0%)
>155 cm	376 (63.8%)	203 (66.1%)	132 (62.3%)	41 (58.6%)
**Maternal Depression (EPDS** ^a^ **)**				
EPDS Score <13	283 (48.0%)	163 (49.8%)	114 (47.7%)	6 (26.1%)
EPDS Score 13–15	72 (12.2%)	41 (12.5%)	28 (11.7%)	3 (13.0%)
EPDS Score >15	200 (34.0%)	115 (35.2%)	77 (32.2%)	8 (34.8%)
Unknown	34 (5.8%)	8 (2.4%)	20 (8.4%)	6 (26.1%)
**Maternal Age at Delivery**				
<20 Years	101 (17.1%)	54 (17.6%)	29 (13.7%)	18 (25.7%)
20 to <35 Years	449 (76.2%)	240 (78.2%)	161 (75.9%)	48 (68.6%)
≥ 35 Years	39 (6.6%)	13 (4.2%)	22 (10.4%)	4 (5.7%)
**Mode of Delivery**				
Vaginal	509 (86.4%)	275 (89.6%)	166 (78.3%)	68 (97.1%)
Cesarean	79 (13.4%)	32 (10.4%)	46 (21.7%)	1 (1.4%)
Unknown	1 (0.02%)	0 (0%)	0 (0%)	1 (1.4%)
**Parity**				
Primipara	208 (35.3%)	109 (35.5%)	72 (34.0%)	27 (38.6%)
Multipara	381 (64.7%)	198 (64.5%)	140 (66.0%)	43 (61.4%)
**Gestational Age at Birth**	36.0 (4.82)	39.4 (1.12)	33.8 (3.30)	27.8 (4.80)
**Sex**				
Male	299 (50.8%)	150 (48.9%)	113 (53.3%)	36 (51.4%)
Female	282 (47.9%)	157 (51.1%)	99 (46.7%)	26 (37.1%)
Unknown	8 (1.4%)	0 (0%)	0 (0%)	8 (11.4%)

^a^EPDS: Edinburgh Postnatal Depression Scale.

### Association of TIs across birth outcomes

**Fig 2:**
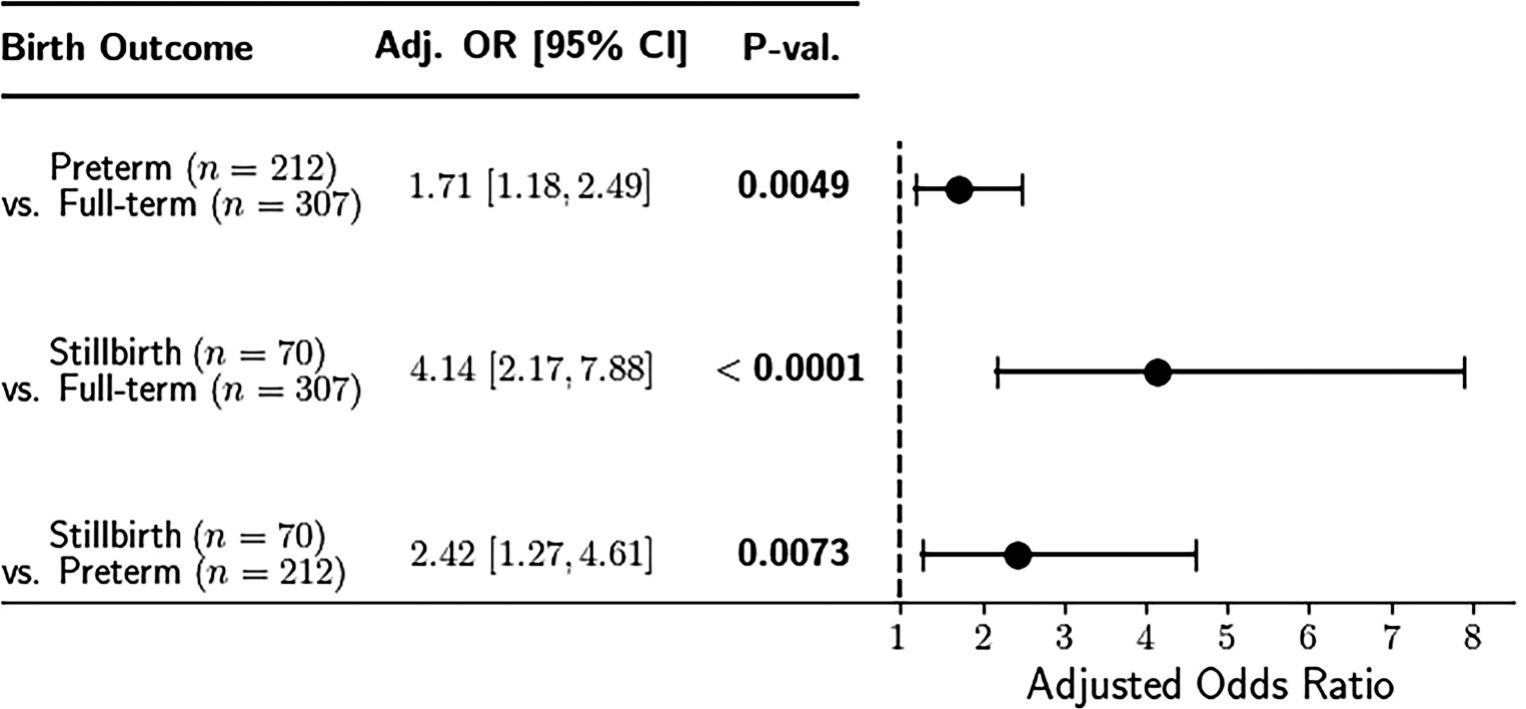
Odds ratios of having TIs in full versus preterm and stillbirth placentas. Forest plot of adjusted odds ratios for identification of placental trophoblast inclusions across three birth outcomes.

Of the 589 placentas examined, at least one TI was observed across the two slides from 108 (35%) of the FTLB placentas, in 103 (49%) of the PTLB placentas, and in 51 (73%) of the SB placentas. Compared to the FTLB placentas, both PTLB placentas and SB placentas were significantly more likely to have TIs. Specifically, the odds of identifying one or more TIs in two slides from PTLB compared to FTLB placentas was 1.74 (95% CI: 1.22, 2.49, *p* = 0.002), while the odds of SB compared to FTLB placentas was 4.95 (95% CI: 2.78, 8.80, *p <* 0.001). After adjusting for maternal age at delivery, education status, marital status, parity, maternal height, and maternal depression, the adjusted odds ratios for PTLB and SB compared to FTLB placentas were 1.71 (95% CI: 1.18, 2.49, *p* = 0.005) and 4.14 (95% CI: 2.17, 7.88, *p <* 0.001), respectively (Fig 2). To account for the possibility that the association between TIs and birth outcome was driven by GA at delivery, we performed an additional linear regression to include GA at delivery as a covariate. This analysis revealed that stillbirths across all GAs at delivery had an average of 2.59 more TIs per case compared to livebirths (full-term and preterm) across all GAs at delivery (95% CI: 1.23, 3.95, *p* = 0.0002).

### Association between TIs and GA at delivery

**Fig 3:**
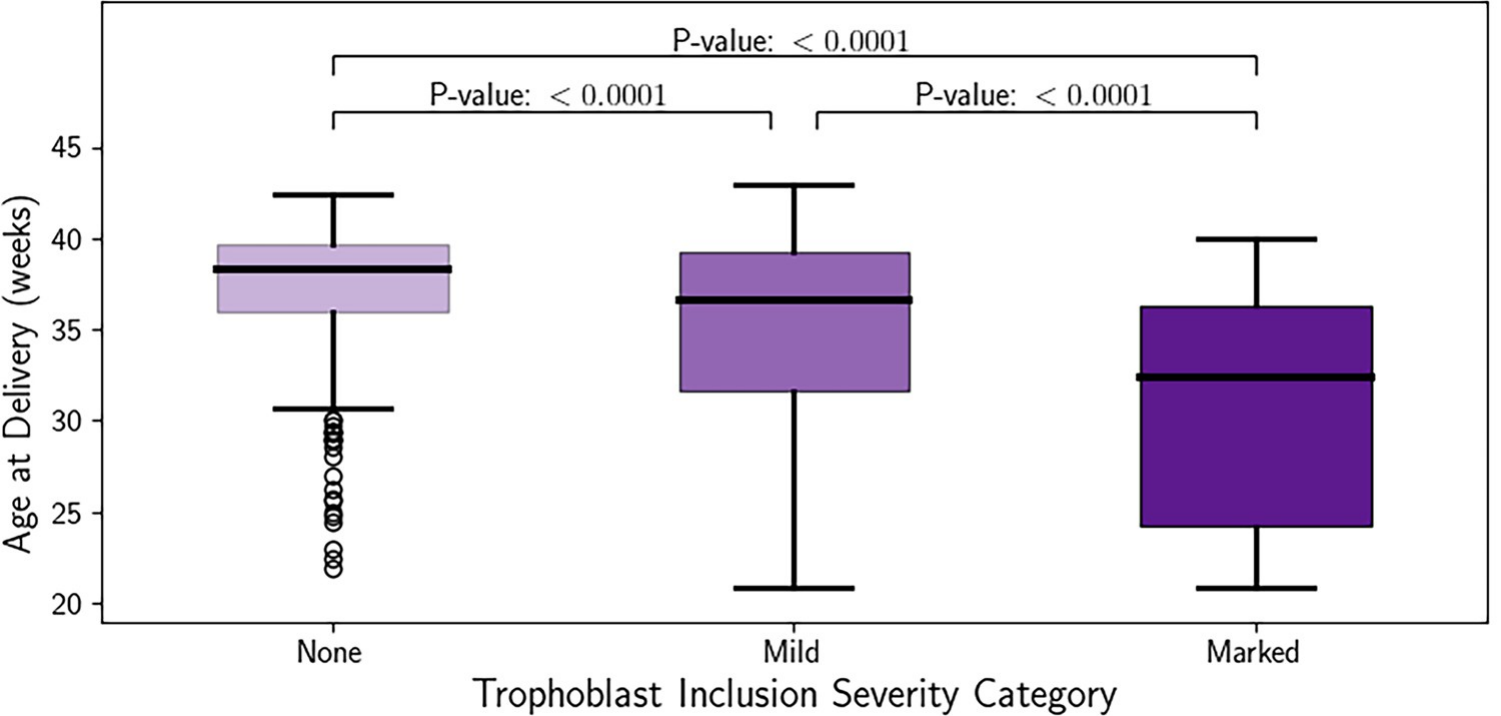
Earlier delivery as a function of TI frequency. Whisker plot of the gestational age at delivery compared to trophoblast inclusion severity category. None (average of 0 TIs per slide), Mild (average of >0–5 TIs per slide), Marked (average of >5 TIs per slide).

Infants with no TIs (‘none’) were delivered between 21.9–42.4 weeks GA, with a median of 38.3 weeks GA and a mean of 37.1 (±3.68) weeks GA at delivery. Infants with ‘mild’ TIs (an average of >0–5 TIs per slide) were delivered between 20.9–43.0 weeks GA, with a median of 36.6 weeks GA and a mean of 35.0 (±5.44) weeks GA at delivery. Infants with ‘marked’ TIs (an average of >5 TIs per slide) were delivered between 20.9–40.0 weeks GA, with a median of 32.4 weeks GA and a mean of 30.8 (±6.63) weeks GA at delivery. To determine if there was a dose-dependent effect of TIs on GA at delivery, we performed an adjusted linear regression. Compared to infants with no TIs, infants categorized as having ‘mild’ TIs were delivered 2.1 weeks earlier (*p* <0.001) (Fig 3). Consistent with a dose-dependent effect, infants categorized as having ‘marked’ TIs were delivered 6.3 weeks earlier compared to those with no TIs (*p*<0.001) and 4.24 weeks earlier than those with ‘mild’ TIs (*p*<0.001).

### Association between TIs and birth characteristics and placental pathologies

**Fig 4:**
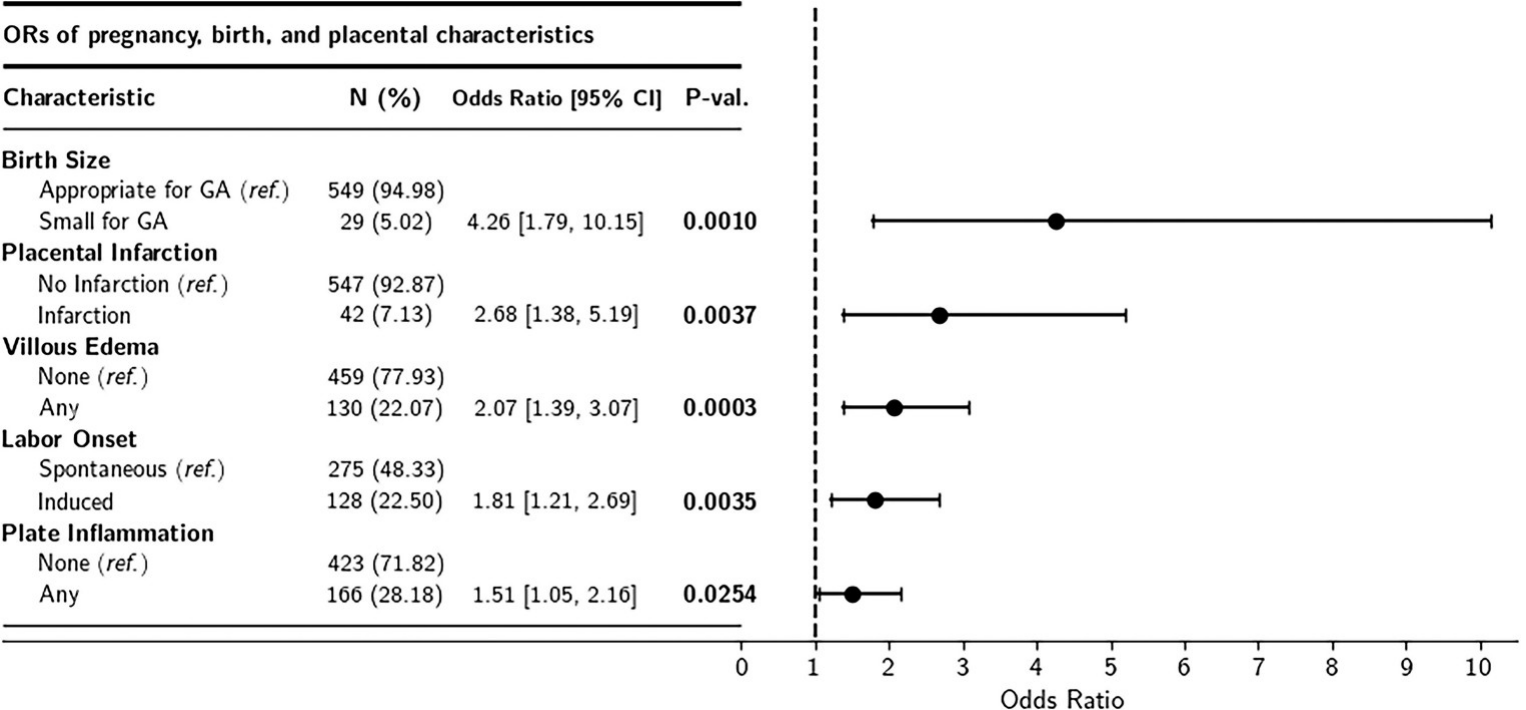
Association of TIs, birth outcomes, and placental pathologies. Forest plot of unadjusted odds ratios for identification of placental trophoblast inclusions across pregnancy, birth, and placental pathology findings.

The presence of villous edema, placental infarction, maternal neutrophilic infiltration of the chorionic plate (plate inflammation), spontaneous labor onset, and neonatal weight that was small for GA were all associated with a statistically significantly higher odds of observing TIs (Fig 4). However, subsequent models that included GA at delivery as a potential mediator revealed that the associations between TIs and villous edema and plate inflammation were no longer statistically significant (*p*’s >0.05). TIs were also not statistically significantly associated with placental weight percentile or any of the other pathologic features examined for this study, regardless of the inclusion of GA in the models (*p*’s >0.05).

## Discussion

### Summary of findings

We report a critical replication and expansion of the previous literature on the role of TIs in placental, pregnancy, and fetal health in a cohort of women with high smoking and alcohol intake exposure. Consistent with an earlier finding [8], the odds of identifying at least one TI across two slides of chorionic villi are significantly higher for placentas from preterm liveborn deliveries. Extending this observation, we demonstrated an even greater odds ratio for placentas containing TIs from stillbirth deliveries. Moreover, we found a significant dose-dependent effect of TIs on GA at delivery such that GA at delivery was inversely associated with TI frequency. To account for the possibility that the association between TIs and birth outcome could be driven solely by GA at delivery, we compared the average number of TIs in placentas from livebirths and stillbirths while controlling for GA at delivery. This analysis revealed that stillbirth placentas had significantly more TIs, even after controlling for GA at delivery, suggesting that TIs are not simply a function of GA at delivery, but rather, there is an independent biological process underlying the association between stillbirth and TIs. In addition to the relationships between TIs, birth outcome, and GA at delivery, we also found significant associations between TIs and a range of pregnancy features including birth weight that was small for GA, induction of labor, as well as placental pathologies including villous edema, placental infarction, and inflammation of the chorionic plate. However, subsequent logistic regression models suggested that GA at delivery mediated the relationship between TIs and villous edema and plate inflammation, though the degree of mediation could not be determined due to sample size limitations.

In a previous study, we did not find an association between TIs and clinical evidence of preeclampsia, gestational diabetes, or hypertension [10]. However, here, we report significantly increased odds ratios for observing TIs in placentas with infarction, villous edema, and migration of maternal neutrophils through the chorionic plate, though these associations may be partially confounded by GA at delivery. Placenta infarction, the death of placental parenchyma most often due to a decrease in maternal perfusion of the placenta, was also associated with the presence of TIs. The connection between maternal perfusion of the placenta and TIs is not obvious. However, placental infarction is often seen in placentas of intrauterine fetal demise. Therefore, this should be examined in future studies. Villous edema is associated with fetal cardiovascular failure, chorioamnionitis, and fetal blood loss [14,21–24]. The association between TIs and villous edema may be due to pathophysiologic changes associated with TIs (such as congestive heart failure secondary to an intrinsic cardiopulmonary defect) or may be indicative of shared factors that both contribute to the development of TIs and that may trigger secondary changes in the pregnancy, such as premature opening of the cervix, which in turn leads to an ascending intraamniotic fluid infection and resultant villous edema [25]. The latter possibility would also explain the association of TIs with chorionic plate inflammation, a marker of the maternal inflammatory response following an ascending infection [26]. Importantly, we found that the relationships between TIs and villous edema and plate inflammation were not statistically significant after including GA at delivery in the model. It remains unclear whether this finding can be attributed to insufficient statistical power due to sample size and study design limitations or to the inter-relatedness of TIs, GA, and these pathologic findings. To fully address the challenge of disentangling these inter-related factors, it would be necessary to compare our cohort to a cohort of placentas derived from healthy, unaffected pregnancies across the same range of gestational ages that we examined.

That none of the other placental pathologic features were associated with TIs, regardless of the inclusion of GA at delivery as a covariate, suggests that the etiology of TIs is independent of the majority of pathologic processes that can impact the placenta. This is consistent with our overarching hypothesis that TIs are a biological marker of genetic and/or developmental abnormality and is consistent with the many studies that have identified TIs in both aneuploid [27–30] and euploid loss placentas [31–35]. As participants of the SPS had a high prevalence of smoking and drinking alcohol during pregnancy, it is also possible that perturbations in the prenatal environment may play a role in the development of TIs.

Our analysis has established an association between TIs and several clinically-relevant outcome measures including preterm birth, stillbirth, small birth weight for gestational age, and specific placental pathologies. Both prematurity and stillbirth are associated with an increased odds ratio of identifying TIs, providing insight into a potential biological process underlying these adverse birth outcomes. Further, this is the first analysis to directly evaluate the relationship between the quantity of TIs and gestational age at delivery. We found that placentas deemed most severe with regards to TI frequency were delivered at a significantly earlier GA than those with no or mild TI frequencies. Given both genetic and *in-utero* factors that may contribute to the incidence of TIs, this relationship may inform clinical management of subsequent pregnancies that are preceded by those in which TIs have been identified.

The associations reported here support the biological plausibility that TIs may indicate dysfunction and/or may play an important role in shaping pregnancy and fetal development. Our data indicate that greater TI severity is associated with lower GA at delivery. However, the observed increased prevalence of TIs in placentas delivered earlier in gestation does not necessitate the same prevalence of TIs in normal undelivered placentas at these same early gestational ages. Earlier literature [7,27–30,36] and our previous studies [4,8–10] clearly associate the presence of TIs with adverse developmental and pregnancy outcomes, which may represent underlying genetic and environmental pathways. The abnormal nature of TIs has been further supported by a study of placentas obtained at early GAs following elective terminations, which found that TIs were present in only 2.8% of the placentas [37].

Given the robust relationships between TIs and the pregnancy, placental, and birth outcomes that we analyzed, future research aimed at understanding the biological mechanisms underlying such outcomes should consider including TIs in the mechanistic pathway. Additionally, further research is needed to determine potential environmental and health-related factors (e.g. tobacco and ethanol exposure), or even placental pathologies that may account for the greater-than-expected proportion of full-term placentas with at least one TI (35% versus previously reported prevalence of 2–8% [7,9,10] noted in the highly exposed cohort we examined for these studies.

### Strengths and limitations

The main strength of our study was the relatively large sample size which was specifically collected for research purposes, rather than relying on information obtained solely for clinical purposes. Therefore, we were able to generate a very detailed and deeply-phenotyped dataset that allowed us to include many potential confounding variables. In a prior publication on the same dataset, we established very high test-retest reliability of the pathologist’s TI counts [6]. This dataset also allowed for replication in a non-US and predominantly non-white study sample. Finally, the sample included an ample representation of placentas from full-term live births, preterm live births, and stillbirths. However, this distribution of cases was by chance, not design—therefore, a potential limitation to our study.

Our initial sample selection was blind to all information regarding the pregnancy and birth outcomes, with the exception of GA at delivery regardless of whether the infant was liveborn or not. Thus, it was not until unblinding of the birth outcomes that we discovered how many stillbirths had been captured in our subsample. Similarly, the sample was not randomly selected based on birth outcome, but rather, was generated based on the specific inclusion criteria previously described. This selection process may have involved unintended bias in sample selection. To address these limitations, it would be beneficial to conduct a prospective analysis of TIs with long-term birth and developmental follow-up. An additional limitation is that we did not have full access to the gross examination of the placenta and rather, were restricted to analyses of two randomly selected slides. This limitation may have impacted our ability to characterize specific features such as the number and location of placental infarcts. Further, genetic analyses were not performed on placentas included in these analyses, and therefore, our data cannot address the biological linkage between genetics, including mosaicism, and the development of TIs. A final limitation of this study is that all slides were reviewed by a single pathologist at one institution, however, it is notable that the pathologist had very high test-re-test reliability [6].

This paper provides foundational associative information regarding the potential role of TIs in a range of placental, pregnancy, and birth processes. The novel associations that we report warrant further investigation in order to understand the complex network of biological mechanisms through which TIs may influence the trajectory and health of a pregnancy. Ultimately, this line of research may provide critical insight that will inform both clinical and research applications.
